# Co-translational import of nuclear-encoded proteins into the chloroplast in *Chlamydomonas reinhardtii*

**DOI:** 10.1093/plphys/kiae310

**Published:** 2024-06-07

**Authors:** Kumari Billakurthi, Naresh Loudya

**Affiliations:** Assitant Features Editor, Plant Physiology, American Society of Plant Biologists; Department of Plant Sciences, University of Cambridge, Cambridge CB2 3EA, UK; Department of Microbiology and Cell Biology, Indian Institute of Science, Bengaluru 560012, India

Photosynthesis constitutes a fundamental process wherein atmospheric inorganic carbon (carbon dioxide) is converted into organic carbon compounds through the absorption of solar energy. This process unfolds within the chloroplast, a green organelle. Chloroplasts are semi-autonomous entities, meaning their development and function hinge on the expression of genes from both the nucleus and chloroplast. Gene expression between the spatially separated cellular compartments, the nucleus and the chloroplast, is highly coordinated, which ensures proper chloroplast development and function (Jarvis and López-Juez 2013).

Unlike chloroplast-encoded proteins, nucleus-encoded chloroplast preproteins are translated in the cytosol and carry signal peptides for their recognition and proper targeting into the organelle. Upon recognition by membrane receptor proteins, the preproteins cross the chloroplast membrane barrier via translocon complexes at the outer and inner envelope of the chloroplast (TOC and TIC). Several protein complexes within the chloroplast are composed of proteins translated from both cytosol and chloroplast: for instance, the TIC import machinery (Kikuchi et al. 2013; Jin et al. 2022), plastid-encoded polymerase (Steiner et al. 2011), and photosystem complexes, among others. In *Chlamydomonas reinhardtii,* a unicellular green algae that contain a single chloroplast, the chloroplast-encoded photosystem complex proteins are synthesized and assembled at a translationally active T-zone (translation zone) near the pyrenoid (Sun et al. 2019).

Co-translational import of proteins occurs when ribosomes engaged in translation are bound to organelles such as mitochondria or the endoplasmic reticulum, facilitating protein import during translation (Williams et al. 2014). Conversely, it has been thought that chloroplast proteins are fully cytosol synthesized and imported post-translationally. It has been unclear whether chloroplast proteins also undergo co-translational import and whether chloroplast protein translation that takes place in the cytosol and chloroplast compartments is spatially coordinated.

In this issue of *Plant Physiology*, Sun, Bakhtiari, and co-workers (Sun et al. 2024) report the enrichment of cytosolic ribosomes in the purified chloroplast fraction of *C. reinhardtii*. This observation prompted an investigation into whether co-translational import of nuclear-encoded proteins occurs in chloroplasts like it does in the endoplasmic reticulum and mitochondria.

To determine if the cytosolic ribosomes in the isolated chloroplasts are physically bound to the chloroplast envelope, the authors performed immunofluorescence microscopy using the cytosolic ribosomal marker protein uL3. The results revealed that in isolated chloroplasts, the uL3 fluorescence signal was concentrated on a discrete basal domain, termed the translation envelope domain (Fig.). This indicates that cytosolic ribosomes are physically associated with the chloroplast envelope. These results were further supported by high-resolution electron tomography.

**Figure.**
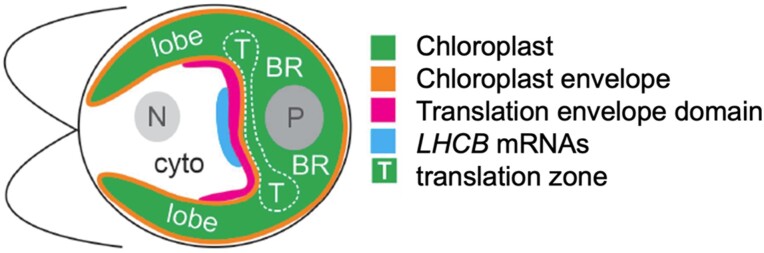
Graphical representation of Chlamydomonas cell shows the spatial organization of translational envelope domain, translation zone, and the localization of light-harvesting complex II proteins (LHCB). BR, basal region; cyto, cytoplasm; N, nucleus; P, pyrenoid. Adapted from (Sun et al. 2024).

To investigate whether the ribosomes in the translation envelope domain are active, Sun et al. (2024) conducted a ribopuromycylation assay to detect the association of nascent polypeptides with the cytosolic ribosomes on the chloroplast envelope. In this assay, isolated chloroplasts were treated with puromycin that mimics tyrosine-tRNA, which enters the ribosome A site and is incorporated into the growing polypeptide chain at the ribosomal P site. This process leads to the premature termination of protein synthesis (David et al. 2012). To prevent the release of puromycin-tagged nascent polypeptides from the site of synthesis, chloroplasts were treated with puromycin under conditions that prevent translation elongation and protein import. Puromycin-tagged nascent polypeptides were then used as markers to map the translation sites with immunofluorescence microscopy with an antibody against puromycin. The fluorescent signal of puromycin was predominantly localized at the translation envelope domain and co-localized with the uL3 marker (Sun et al. 2024). These results demonstrate that the cytosolic ribosomes associated with the chloroplast envelope are translationally active.

The translation envelope domain is spatially coordinated with the T-zone in the chloroplast, which is the site for the biogenesis of chloroplast-encoded photosystem complex proteins such as PsbA (Sun et al. 2019). Moreover, using fluorescent in situ hybridization of mRNAs encoding light-harvesting complex II proteins, the authors confirmed that the translation envelope domain is bound by mRNAs encoding chloroplast localized proteins (Fig.).

In summary, this study sheds light on the co-translational import of nuclear-encoded chloroplast proteins in *C. reinhardtii*. Furthermore, it reveals that the translation envelope domain and the T-zone are closely aligned on the cytosolic and stromal side of the chloroplast envelope, respectively (Fig.). The authors propose that the spatial coordination facilitates the localization of newly synthesized subunits to the T-zone, where chloroplast-encoded proteins are translated, for the assembly of the photosynthesis complexes.

This work also opens several important questions. Does the spatial coordination of translation apply to other chloroplast protein complexes, such as the TIC complex, which is composed of both nucleus-encoded and chloroplast-encoded proteins? What is the full profile of cytosolic mRNAs translated on the translation envelope domain? How are ribosomes associated with the chloroplast translation envelope domain? Does this mechanism also occur in land plants that contain multiple chloroplasts per cell? How is this mechanism regulated during altered or unfavorable growth conditions?
